# Vibrational Study (Raman, SERS, and IR) of Plant Gallnut Polyphenols Related to the Fabrication of Iron Gall Inks

**DOI:** 10.3390/molecules27010279

**Published:** 2022-01-03

**Authors:** Alba Espina, Santiago Sanchez-Cortes, Zuzana Jurašeková

**Affiliations:** 1 Department of Biophysics, Faculty of Science, Pavol Jozef Safarik University, 040 01 Kosice, Slovakia; alba.espina.garcia@student.upjs.sk; 2 Instituto de Estructura de la Materia (CSIC), Serrano, 121, 280 06 Madrid, Spain; s.sanchez.cortes@csic.es; 3 Center for Interdisciplinary Biosciences, Technology and Innovation Park, Pavol Jozef Safarik University, 040 01 Kosice, Slovakia

**Keywords:** polyphenols, gallnut, iron gall inks, Raman, FTIR, SERS

## Abstract

FT-Raman, FTIR, and SERS spectra of the structurally related gallnut polyphenols tannic acid, gallic acid, pyrogallol, and syringic acid are reported in this work aiming at performing a comparative assignation of the bands and finding specific marker features that can identify these compounds in complex polyphenol mixtures. Tannic and gallic acids are the principal components in oak gallnuts, and they can be found in iron gall inks. The different functional groups existing in these molecules and their spatial distribution lead to slight changes of the vibrations. The Raman spectra are dominated by bands corresponding to the ring vibrations, but the substituents in the ring strongly affect these vibrations. In contrast, the FTIR spectra of these molecules are dominated by the peripheral oxygen-containing substituents of the aromatic ring and afford complementary information. SERS spectroscopy can be used to analyze trace amounts of these compounds, but the spectra of these polyphenols show strong changes in comparison with the Raman spectra, indicating a strong interaction with the metal. The most significant modification observed in the SERS spectra of these compounds is the weakening of the benzene *8a* ring vibration and the subsequent intensification of the *19a* mode of the benzene ring. This mode is also more intense in the FTIR spectra, and its intensification in the SERS spectra could be related to a drastic change in the molecular polarizability associated with the interaction of the polyphenol with the metal in Ag NPs.

## 1. Introduction

Phenolic compounds generally are the most abundant secondary metabolites in plants, demonstrating many beneficial properties/activities. However, despite their importance and characteristic spectral features, there have been few works on correctly identifying and quantifying them. The phenolic compounds existing in plant galls deserve special attention. Galls are abnormal growths of tissue in certain parts of the plant in response to a specific stimulus from an invading organism [1]. These formations contain specific nutrients for insects and a high level of polyphenolic compounds that can serve to protect the hosted insect from the attack of parasitoids [2,3]. Oak galls contain a large amount of tannic acid and lower amounts of gallic and ellagic acids [4]. These galls have been the principal ingredient used in the preparation of iron gall inks (IGIs) because one of the main chemical properties of these polyphenols is the high affinity to link metals, leading to the formation of metal complexes.

Tannic acid (TA) is a natural polyphenol extracted from various plants with a structure composed of a central glucose molecule esterified at all five hydroxyl moieties with two gallic acid molecules. TA is water-soluble and possesses antioxidant, antibacterial, and anticarcinogenic properties; thus, it is used in a wide range of food and biomedical applications [5,6,7,8]. Gallic acid (GA) is involved in many biochemical processes and reactions and is of great importance in the environment thanks to its antioxidant and prooxidant properties [9]. It also has anticancer, antiviral, antibacterial, and allelopathic effects [10,11,12]. Furthermore, pyrogallol (PY) and syringic acid (SA) are other natural polyphenols naturally existing in plant gallnuts [13,14].

IGIs were historically the most important and common writing and drawing material until the beginning of the 20th Century [15,16,17]. The reasons for that are their simple preparation, the good quality of the written records, their indelibility, as well as not clogging writing pens and brushes [18]. Nevertheless, during the 20th Century, they were progressively replaced by modern writing media based on synthetic dyes and colorants, which do not show corrosive properties [19]. Anyway, a significant part of the human historical and cultural heritage has been recorded with IGIs. Hence, the analysis of IGIs can reveal useful information about the origin, antiquity, and authenticity of historical documents, as well as their preservation.

The complexity of studying IGIs’ chemistry lies in the numerous recipes and ingredient sources used, resulting in differing compositions and component proportions. Depending on their composition, many IGIs have a corrosive nature and may be subjected to redox reactions, which may lead to a color change. These processes induce the deterioration of many manuscripts and artworks, while some of them are surprisingly in excellent condition. The identification of IGI on an object, as well as the analysis of its conservation/deterioration state is important not only from a historical and curatorial perspective, but also because of preservation and the application of appropriate conservation treatments.

Historical IGI recipes typically consist of four basic ingredients: gall extracts (mainly tannic or gallic acids either from plant gallnut extract or, in later years, as pure compounds), ferrous sulfates (vitriols), gum Arabic, and a liquid medium (water, wine, vinegar, etc.) [20]. The polyphenol composition of gallnuts is very important to determine the nature of the resulting inks. Therefore, the analysis of these compounds could afford much information about the study of the ink’ origin, its chemical content, and its stability over time.

Raman spectroscopy (RS) and surface-enhanced Raman scattering (SERS) have proven to be suitable, efficient, non-destructive techniques and even applicable in situ techniques for the analysis of phenolic compounds. Moreover, they provide very specific structural fingerprint information of a wide range of materials used in different cultural heritage artifacts [21,22]. The SERS technique can be used for in situ analysis of highly fluorescent molecules in cultural heritage artifacts of interests, with the additional advantage of its high sensitivity, which can achieve results comparable to those obtained through commonly used chromatographic methods [23]. In addition, further studies have shown the non-destructiveness or micro-destructiveness of SERS [24,25,26,27,28,29], since the samples do not undergo any chemical pre-treatment. These and other results have demonstrated that SERS spectroscopy appears to be a suitable technique for analyzing artworks [30,31,32,33]. Specifically, Raman spectroscopy has been successfully employed in the in situ analysis of pigments and colored inks contained in illuminated manuscripts. Nevertheless, despite the clear advantages of Raman spectroscopy and SERS, there are few studies dealing with the in situ characterization of manuscripts by Raman spectroscopy or SERS, and it is still not a regular technique used in the analysis of IGIs [16,34,35,36,37].

The characterization of IGIs by Raman spectroscopy requires a previous analysis of the polyphenols integrated in gallnuts. Although this kind of study has been performed for different phenolic compounds [38,39], a comprehensive comparative analysis is necessary to distinguish between the main vibrational features in a mixture of such gallnut compounds. Raman spectroscopy alone is not always able to distinguish the sources of specific functional groups, especially in such complex systems as (paper-based) artifacts. Therefore, it is often necessary to also use other techniques, such as complementary FTIR spectroscopy or X-ray fluorescence measurements [40,41].

**Figure 1:**
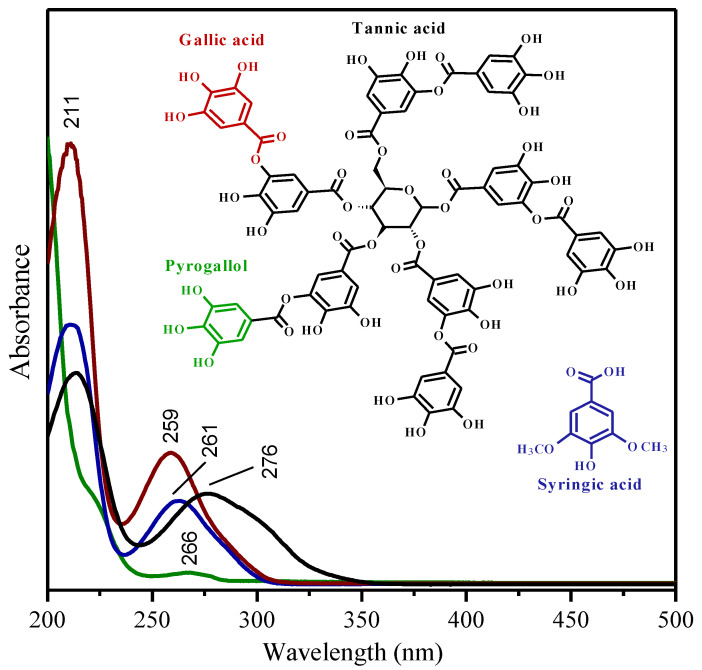
UV-Vis absorption spectra of the studied polyphenolic compounds. The inset shows the molecular structure of the molecules. It is worth noting that the molecular structure of gallic acid and pyrogallol can be found in the structure of the tannic acid molecule.

We present in this paper a vibrational study by Raman spectroscopy, FTIR and SERS, together with an absorption UV-Vis study, of four fundamental polyphenols existing in plant gallnuts: tannic acid, gallic acid, pyrogallol, and syringic acid (Figure 1). The SERS spectra of TA, PY, and SA are reported here for the first time, and this technique could be used to detect these polyphenols at very low concentrations, avoiding the problem of high fluorescence. The goal of this work was to perform a comparative analysis of the vibrational modes of these compounds and the identification of spectral marker bands that could allow the proper assignment of the vibrational modes and to follow how the spectra of these compounds can be modified in the presence of a metallic surface, such as the surface of silver in plasmonic nanoparticles (NPs). Indeed, this assignation will also allow the proper identification of these compounds in vegetal or food materials, as well as other materials related to cultural heritage such as manuscripts, while SERS can afford vibrational spectra from minute quantities of the analyzed material. We chose the above polyphenols because their comparison is essential to understand the influence of the different chemical groups on the resulting vibrational spectra. Furthermore, this is a preliminary step in the study of the different effects of the complexation with iron and copper regarding the fabrication of IGIs.

## 2. Results and Discussion

### 2.1. UV-Visible Absorption Spectra

UV-Vis spectroscopy is a very suitable technique for the study and quantification of phenolic compounds [20,42,43]. These colorless compounds do not show absorption features in the visible region of the electromagnetic spectrum. However, phenolic substances can strongly absorb UV light. Therefore, certain phenolic compounds can still lead to absorption features also in the visible range, displaying a color. Pyrogallol, having three hydroxyl groups attached to the aromatic ring, presents only a weak absorption maximum at 266 nm and a medium shouldering band at 222 nm (Figure 1).

On the contrary, the UV-Vis absorption spectrum of GA in an aqueous solution shows two characteristic phenol bands appearing at 211nm and 259 nm, corresponding to the 1L_a_ and 1L_b_ bands, respectively, originating from π→π^*^ transitions [44,45]. In the case of SA, the latter can be observed as slightly red-shifted (to 261 nm) because of the methyl substitution of two of the three phenolic hydroxyl groups, i.e., because of the larger conjugated system. For tannins, despite the degree of polymerization of these polyphenolic structures, the absorption features remain the same with a predominant absorption band at around 280 nm. In particular, at neutral pH, the absorption spectrum of the TA aqueous solution exhibits two peaks at 216 nm and 276 nm with a medium shouldering band at 308 nm (Figure 1) assigned to its neutral form, as was previously reported elsewhere [46,47,48].

### 2.2. Raman and IR Spectra

**Figure 2:**
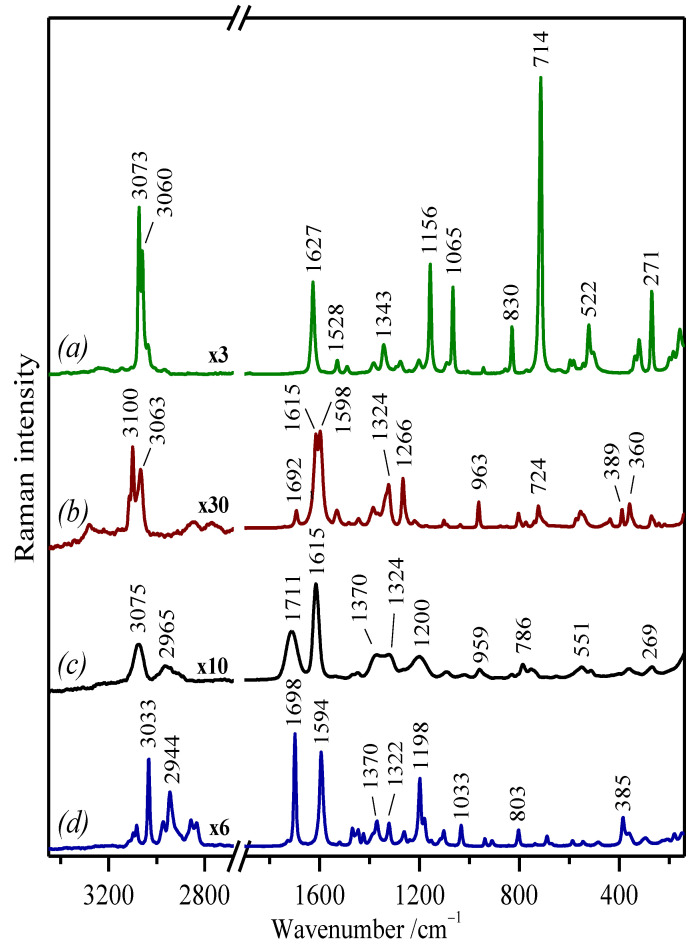
FT-Raman spectra of the studied polyphenolic compounds in their solid state: (**a**) pyrogallol; (**b**) gallic acid; (**c**) tannic acid; (**d**) syringic acid. Raman spectra were normalized to the bands at 1627–1594 cm^−^^1^. Excitation line: 1064 nm.

Raman spectroscopy (RS) allows us not only to identify and characterize the selected compounds, but also to monitor, for example, the deprotonation of the molecules, their interactions with metal ions, as well as different structural changes that occur during these processes. Since the four phenolic compounds analyzed here exhibit a certain fluorescence emission in the UV-Vis region, we registered the FT-Raman spectra in their solid state (Figure 2).

As expected, spectral differences can be observed, but at the same time, common spectral features can also be found in the recorded spectra. This allowed us to compare them and, consequently, to carry out an assignment of their vibrational modes. Performing a complete and detailed vibrational assignment is not a simple task. In fact, no satisfactory assignment of the bands was found in the literature, although the Raman spectra of many phenolic compounds have been already published [38,46,49,50,51,52,53,54,55]. Furthermore, the comparison of the IR and Raman spectra also serves as an additional assistance toward a more accurate assignment, since they are complementary due to the different selection rules applied in these techniques, which give rise to vibrational bands with different relative intensities. Thus, a vibrational assignment was performed on the basis of the recorded vibrational spectra, as well as the results found in the literature for the studied molecules, their derivatives, as well as other structurally related molecules [46,53,55,56,57,58,59]. The experimental IR and Raman frequencies for various modes of vibration were assigned and are presented in Table 1.

In general, all polyphenols showed a similar spectrum below 1000 cm^−^^1^ (in the “fingerprint” region) owing to the similar skeletal structure; however, the exception was PY’s Raman spectrum. In contrast, the spectral region from 2000–1000 cm^−^^1^ displayed a higher diversity. This was not the case for the region corresponding to the C=O and C=C stretching vibrations (ν(C=O) and ν(C=C)) seen in the 1710–1530 cm^−^^1^ region, where some similarities were found between these molecules, with the exception of PY, which has no ν(C=O) functional group. However, the main differences between them were found in the 1400–1000 cm^−^^1^ region where OH deformations (δ(O–H)) and ν(C–O) motions appear, and this was because of the different substitution and distribution of the functional groups (OH, OCH_3_) in the studied molecules. Finally, the region from 4000–2800 cm^−^^1^ is characteristic of the stretching vibrational modes associated with the groups containing hydrogen (O–H, C–H), which is also variable according to the substitution pattern of the aromatic rings.

The Raman spectrum of the PY molecule differs the most (compared to three other molecules) regarding especially the intensity of the observed bands (Figure 2a). Firstly, an intense Raman band at 3073 cm^−^^1^ assigned to the aromatic C–H stretching vibrations (ν(C–H)) is visible. GA and TA do not differ too much concerning the frequency of the ν(C–H) bands. In particular, the bands at 3100/3063 cm^−^^1^ and 3075/2965 cm^−^^1^ can be seen in the Raman spectra of GA and TA, respectively (Figure 2b,c). Nevertheless, the Raman intensities of the ν(C–H) bands are relatively weaker in these two polyphenols. Furthermore, as expected, the Raman spectrum of SA showed a higher number of bands at this high-wavenumber edge of the spectrum due to the presence of two methyl groups, which give rise to the symmetric and asymmetric stretching modes of the O–CH_3_ group observed at 2834 cm^−^^1^ and 2857 cm^−^^1^ and at 2944 cm^−^^1^ and 2973 cm^−^^1^, respectively. The stretching at 3033 cm^−^^1^ is the most intense ν(C–H) band among these polyphenols.

The bands attributed to ring C–C stretching appear in the region between 1400 cm^−^^1^ and 1650 cm^−^^1^. In the Raman spectrum of PY, the benzene C=C stretching *8a* mode appears at a relatively high position: 1627 cm^−^^1^ (Figure 2a). In the case of GA, a doublet at 1615 cm^−^^1^ and 1598 cm^−^^1^, assigned to the phenyl stretching vibrations coupled with the δ(O–H) and CH in-plane bending modes, is visible. It was seen that the observed doublet is characteristic of the GA crystals present in the monomer form [56,60,61]. Because of the concurrent presence of the band at 1692 cm^−^^1^, we can also suppose the presence of the dimeric form of gallic acid. However, the monomer dominates the Raman spectrum of GA in its solid state, as deduced from the recorded FT-Raman spectrum.

The high position of the *8a* stretching vibration in PY (1627 cm^−^^1^) compared to the other polyphenols can be attributed to absence of the conjugation of the C=O bond with carbon double bonds of the phenyl ring that occurs in the other polyphenols. The ν(C=O) of the carboxylic group is in the Raman spectrum of GA, visible at 1692 cm^−^^1^ as a very weak band. On the contrary, the band at 1698 cm^−^^1^ in the Raman spectrum of SA is the strongest ν(C=O) band of this series (Figure 2d). The high intensity of this band, compared to the equivalent one in GA, points out that this molecule does not form dimmers, as in the case of GA.

TA displays a medium-intense and broad band at 1711 cm^−^^1^ assigned to the conjugated ν(C=O) vibration in the ester group (Figure 2c). The broadening of this band is due to the numerous carbonyl groups found in the structure of TA, as well as the existence of different intra- and inter-molecular hydrogen bonds. A second strong band at 1615 cm^−^^1^ in the Raman spectrum of TA can be also seen, which is assigned to the C=C stretching *8a* mode at a similar position as GA. In general, TA presents very broad spectral bands due to the different possible structures existing in the molecule.

In the next region, two intense bands at 1156 cm^−^^1^ and 1065 cm^−^^1^ can be seen in the Raman spectrum of PY (Figure 2a). They are related to the C–H in-plane bending vibrations, ring-deformation in-plane bending modes, and C–O stretching vibrations [46,53]. Finally, the fingerprint zone of the PY Raman spectrum is dominated by the intense band at 714 cm^−^^1^, which is attributed to the ring-breathing mode [53].

The major differences between the three molecules (GA, SA, and TA) can be found in the 1500–1000 cm^−^^1^ region, where the C–H and O–H bending modes, as well as C–O stretching motions appear, because of the different functional groups found in the structure of these molecules. In addition, bands assigned to aromatic ν(C–C) and δ(C-C–H) also appear in this region, while the ν(C–O) modes mainly appear in the 1300–1200 cm^−^^1^ interval. SA shows many bands in this spectral region because of the presence of the C–H bending modes of the methyl groups, showing the most intense band at 1198 cm^−^^1^, assigned mainly to the ring δ(CH) vibrations (Figure 2d). GA displays two relatively intense bands at 1324 cm^−^^1^ and 1266 cm^−^^1^ ascribed to the ν(C–C), ν(C–O), and δ(C–OH) modes (Figure 2b). The spectrum of TA displays similar bands, but also displaying a general broadening of the different vibrational modes (Figure 2c). This is also typical for complex molecular systems where numerous hydrogen bonds occur [62].

Since the TA polymer includes in its structure many GA units, the comparison between the Raman spectra of both molecules is interesting in order to understand the effect of polymerization in GA, aiming at the discrimination between these two molecules in IGIs. It is a very well-known fact that TA can be degraded under certain conditions, such as an acidic pH or by oxidation of the polymer [63]. Therefore, the finding of spectral markers associated with this degradation could be very helpful in the analysis of the chemical status. The main differences found between the Raman spectra of both molecules in their solid state are related to a general broadening of the bands, as already mentioned, and the presence of the three main bands in TA at 1711 cm^−^^1^, 1370 cm^−^^1^, and 1200 cm^−^^1^ (Figure 2c), attributed to the ester groups existing in TA. In addition, the presence of characteristic bands at 1692 cm^−^^1^, 1530 cm^−^^1^, 1266 cm^−^^1^, and 724 cm^−^^1^ in the GA Raman spectrum (Figure 2b) can be related to the carboxylic group in the non-polymerized molecule.

**Figure 3:**
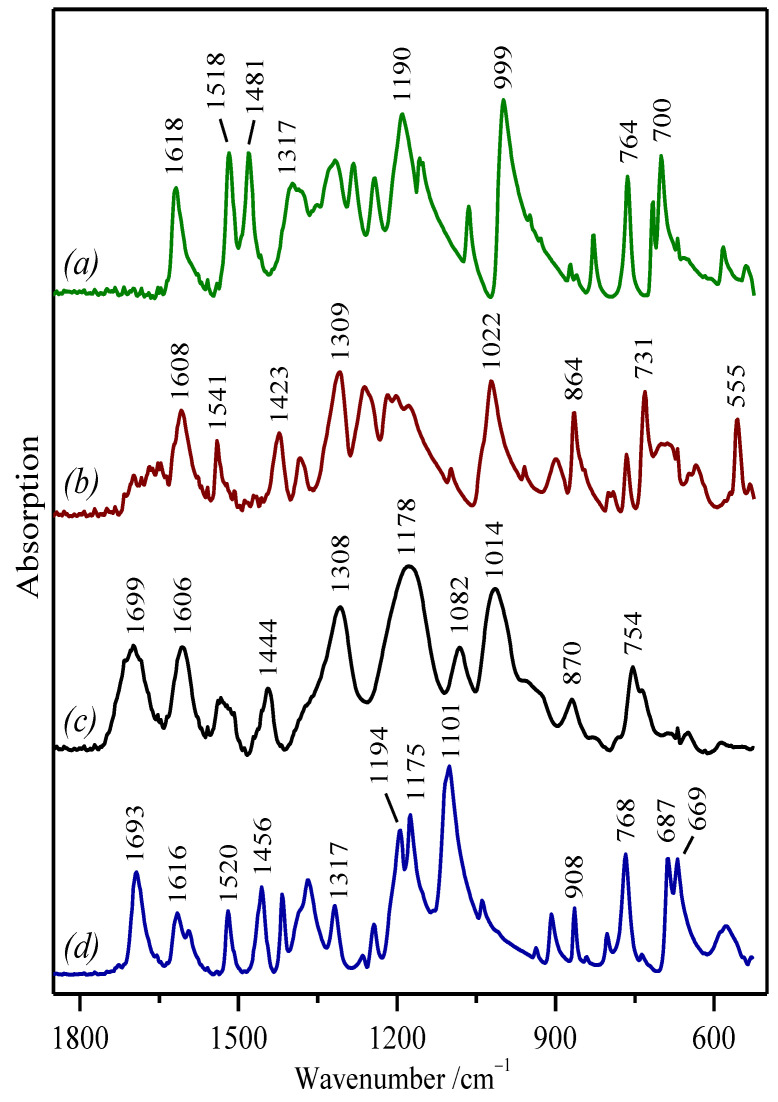
ATR-FTIR spectra of the studied polyphenolic compounds in their solid state: (**a**) pyrogallol; (**b**) gallic acid; (**c**) tannic acid; (**d**) syringic acid.

To complete the vibrational features of the studied molecules, we also recorded their FTIR spectra in the region from 2000–520 cm^−^^1^ (Figure 3). Since the molecules have no center of symmetry, most of their fundamental vibrations are active in both the Raman and IR spectra, whereas the FTIR spectra of all four molecules show greater similarity than their Raman spectra. As expected, the IR spectra of phenolic compounds show common spectral bands associated mainly with the aromatic six-membered rings and phenol moieties.

The FTIR spectra of these molecules are dominated by strong broad bands in 1200–1000 cm^−^^1^ attributed to ν(C–O), ν(C–C), and in-plane δ(C–H). In general, the vibrations containing the in-plane δ(C–H) vibration appear in the range of 1450–1000 cm^−^^1^, while the vibrations including the out-of-plane δ(C–H) motions are stronger in the FTIR spectra and appear in the 1000–750 cm^−^^1^ range in aromatic compounds (Table 1). In addition, the O–H in-plane bending motions may couple with C–H bending vibrations and ring-stretching vibrations, leading to very broad bands appearing in the region of 1420–1300 cm^−^^1^. Besides, broad absorption bands appearing in the region 710–520 cm^−^^1^ are generally associated with the out-of-plane bending vibrations of the O–H group, and this is characteristic of the spectra of alcohols and phenols [46,53].

SA displays a characteristic series of very narrow medium-intense bands at 1456–1317 cm^−^^1^ attributed mainly to in-plane deformations and associated with the presence of the methyl groups in its structure (Figure 3d). On the contrary, the GA and TA FTIR spectra display intense absorption bands at 1309 cm^−^^1^ and 1308 cm^−^^1^, respectively (Figure 3b,c), which are related to the coupled vibrations of the ring ν(C–C) and the carbonyl ν(C=O), together with the contribution from the δ(C–H) and δ(C–OH) vibrations [56].

In the FTIR spectra of TA and SA, the band assigned to the ν(C=O) vibrations is observed at 1699 cm^−^^1^ and 1693 cm^−1^, respectively (Figure 3c,d). In the case of GA, there is just a weak broad and not well-defined band in this spectral range due to the existence of dimmers that partially quench the activity of the ν(C=O) mode in –COOH dimer vibrations (Figure 3b). As expected, PY does not possess this vibration (band in its spectrum; Figure 3a) due to the absence of the carboxyl group in its structure.

While in the Raman spectra, the ring stretching vibrations in polyphenols are very prominent and highly characteristic of the aromatic rings (these vibrations appear in the range of 1650–1200 cm^−^^1^), in the FTIR spectra, these vibrations are less intense than those involving oxygen functional groups. For instance, the *8a* benzene ring vibration appears at 1618 cm^−^^1^ in PY (Figure 3a). However, PY shows two more intense and characteristic bands observed at 1518 cm^−^^1^ and 1481 cm^−^^1^ attributed to δ(C–OH) coupled to ring stretching motions that correspond to the *19a* and *19b* ring vibrations [53]. GA shows the *8a* benzene ring band at 1608 cm^−^^1^ and a new band at 1541 cm^−^^1^, also corresponding to δ(C–OH) motions coupled to ring-stretching vibrations (Figure 3b). In the TA FTIR, the *8a* benzene ring spectrum is downshifted to 1606 cm^−^^1^, and an equivalent broad band centered at 1530 cm^−^^1^ appears (Figure 3c). In the case of SA, the ring stretching appears at 1616 cm^−^^1^ with a shoulder at 1595 cm^−^^1^, and in addition, bands at 1520 cm^−^^1^ and 1456 cm^−^^1^ attributable to δ(C–OH) and δ(CH_3_) are also seen (Figure 3d).

As mentioned above, the ν(C–O) stretching vibrations also coupled with the adjacent ν(C–C) modes lead to a strong band in the 1260–1000 cm^−^^1^ region. Since these bands occur at different positions depending on the structure of the analyzed compounds, they could be good spectral markers for the identification of each polyphenol. For instance, intense bands can be observed at 1190 cm^−^^1^ and 999 cm^−^^1^ in the PY FTIR spectrum; in the GA FTIR spectrum, this can be observed at 1022 cm^−^^1^; the TA FTIR spectrum shows broad bands at 1178 cm^−^^1^ and 1014 cm^−^^1^; finally, a doublet at 1194 cm^−^^1^/1175 cm^−^^1^, as well as a very strong band at 1101 cm^−^^1^ are seen in the FTIR spectrum of SA. The latter can also be assigned to the ether C–O–C bond existing in this molecule [64].

Furthermore, the analysis of the fingerprint zone, where out-of-plane bending vibrations of the O–H group mainly appear, reveals the existence of characteristic marker bands associated with each polyphenol. For instance, PY shows two intense bands at 764 cm^−^^1^ and 700 cm^−^^1^; in the GA spectrum two bands, at 731 cm^−^^1^ and 555 cm^−^^1^, are visible; TA with its complex structure gives rise to a broad and not so intense band at 754 cm^−^^1^ with a shoulder centered at 737 cm^−^^1^; finally, SA displays a series of very intense bands at 768 cm^−^^1^ and 687 cm^−^^1^/669 cm^−^^1^.

### 2.3. SERS Spectra

**Figure 4:**
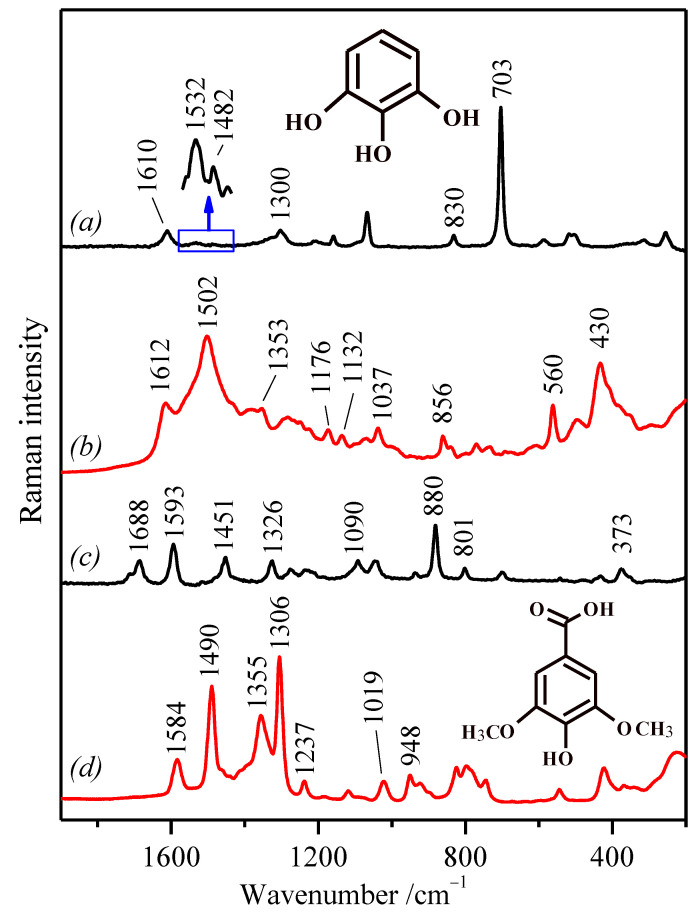
(**a**) Raman spectrum of PY in aqueous solution (20 mg/mL); (**b**) SERS spectrum of PY (at concentration 10^−5^ M) in AgC NPs; (**c**) Raman spectrum of SA in ethanol solution (20 mg/mL) after subtracting the ethanol spectrum; (**d**) SERS spectrum of SA (at concentration 10^−5^ M) in AgH NPs. Excitation line: 785 nm.

**Figure 5:**
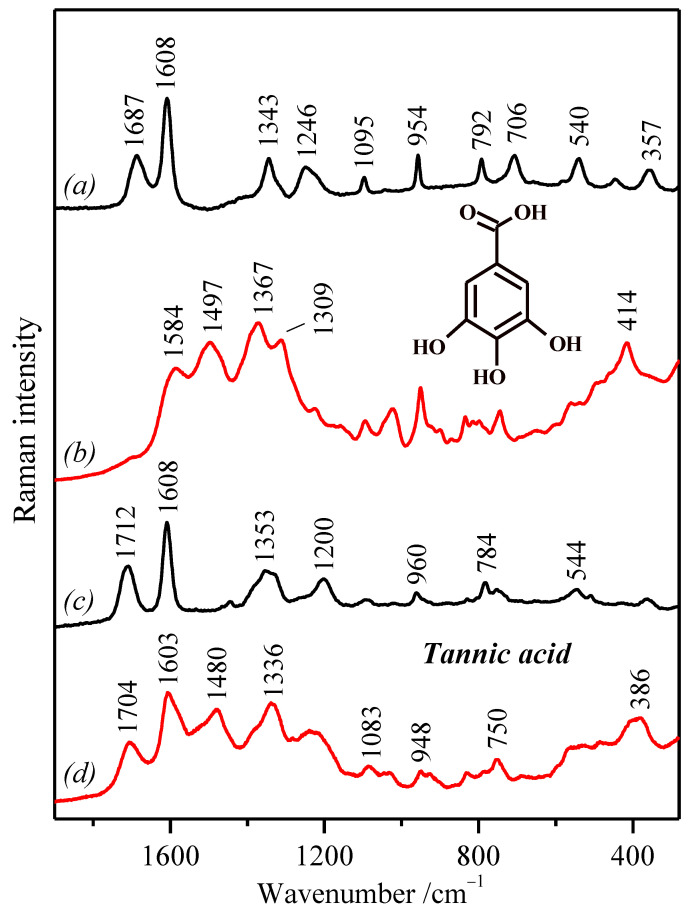
(**a**) Raman spectrum of GA in aqueous solution (20 mg/mL); (**b**) SERS spectrum of GA (at concentration 10^−5^ M) in AgC NPs; (**c**) Raman spectrum of TA in aqueous solution (20 mg/mL); (**d**) SERS spectrum of TA (at concentration 10^−5^ M) in AgC NPs. Excitation line: 785 nm.

The corresponding SERS spectra of the analyzed polyphenols (Figure 4 and Figure 5) show significant changes in comparison with the Raman spectra of the polyphenols both in the solid state (Figure 2) and in aqueous or ethanol solution. The Raman spectra of polyphenols in aqueous solution (in the case of SA in ethanol) are also shown in Figure 4 and Figure 5 for comparison with the corresponding SERS spectra. In solution, these polyphenols only show slight spectral differences in relation to the solid state attributable to the different molecular states with respect to the formation of intra-molecular and inter-molecular hydrogen bonds in the solid state and the interaction with the solvent.

The SERS spectra were obtained by using AgC NPs. On the contrary, no SERS spectra could be obtained on AgH NPs except for the SA molecule (Figure 4d). However, the AgC colloid may show bands of the residual citrate ions used for preparing the colloid, which can overlap the bands of the analyte. The higher activity of AgC NPs to render intense SERS spectra is related to the fact that the citrate residual species, still adsorbed onto the metal surface, and its oxidation products can better interact with the –OH groups of polyphenols through the establishment of H-bonds between –COOH in citrate and the –OH groups in the phenols [65].

In general, the SERS spectra are dominated by broad bands that may corroborate the formation of H-bonds between the adsorbed molecules and the organic molecules covering the NPs. Moreover, the big changes observed between the Raman spectra in solution and the SERS point out the occurrence of strong polyphenol structural modifications as a consequence of their interaction with the metal surface. Another reason that may contribute to the broadening of bands is the deep structural changes that these polyphenol molecules may undergo because of a possible polymerization, leading to more complex structures, as we also reported in previous works [58,66].

At first glance, the adsorption of the acidic polyphenols (GA and SA) on Ag nanoparticles induces the ionization of carboxylate groups, which can be deduced from the weakening of the bands at 1688 cm^−^^1^ and 1687 cm^−^^1^, for SA and GA, respectively, of the solution (Figure 4c and Figure 5a). In addition, a remarkable weakening of the *8a* mode is observed in all the cases. Another important feature in the SERS of all polyphenols is the intensification of a band in the 1505–1475 cm^−^^1^ region, attributed to the *19a* mode of the benzene ring [67,68,69], which seems to be enhanced regarding the same weak band in aqueous solution upon the interaction of these polyphenols with the metal surface (see the expanded spectrum in Figure 4a in the 1450–1550 cm^−^^1^ region). This mode is more intense in the FTIR spectra, due to the fact that the corresponding vibration implies a strong variation of the molecular dipolar moment, while the variation of polarizability is rather weak. However, the interaction with the metal at the surface seems to induce a structural change of the molecule in such a way that leads to an increase of the variation of the polarizability associated with the *19a* mode. Furthermore, another general characteristic of the SERS spectra is the enhancement of a new broad band, which appears with a high intensity in the 435–410 cm^−^^1^ region in the SERS spectra of PY, GA, and SA (Figure 4b,d and Figure 5b), while in TA, it appears at slight lower wavenumbers (386 cm^−1^) (Figure 5d).

It is worth noting the singularity of the SA SERS spectrum (Figure 4d) regarding the observation of sharp and intense bands, unlike the SERS spectra of the other three molecules (GA, TA, and PY). This result can be related to the presence of the methoxy groups in its structure. These groups may exert an influence on the natural tendency of polyphenols to polymerize, and for this reason, sharper bands could be observed. The strong band observed at 1306 cm^−^^1^, attributed to the ν(C–O) motion coupled to ring stretching, seems to be evidence of the preservation of the original structure of this molecule and the absence of any polymerization.

In contrast, the SERS spectrum of PY (Figure 4b) displays strong changes regarding the aqueous solution with a new strong band at 1502 cm^−^^1^ together with the intensity decrease of the *8a* vibration. However, the most important change in this molecule is the complete disappearance of the ring-breathing band seen in the aqueous solution at 703 cm^−^^1^ (Figure 4a). Besides, new strong bands in the low wavenumber region, i.e., at 560 cm^−^^1^ and 430 cm^−^^1^, appear likely associated with the deep structural changes undergone by this molecule upon adsorption on the surface.

The SERS spectrum of GA also displays a great deal of changes, which indicate a deep structural change of this polyphenol on Ag NPs (Figure 5b). There are hints that point to a direct interaction of GA carboxylate groups with silver, specifically the strong weakening of the 1687 cm^−1^ band in aqueous solution (Figure 5a), attributed to carboxylic acid, and the enhancement of the band at 1367 cm^−1^ in the SERS spectrum (Figure 5b), attributed to the carboxylate group in the gallate ion.

In contrast to the other analyzed polyphenols, the SERS spectrum of TA (Figure 5d) is the one that displays a lower difference in comparison to the corresponding Raman spectrum (Figure 5c). The main difference is the new band at 1480 cm^−1^, associated with the *19a* ring mode due to the interaction of the phenol groups with the metals, which is general for all the other polyphenols. The higher correspondence with the Raman spectrum in solution indicates that TA may undergo a lower structural transformation on the silver surface due to its higher size and the fact that it is an already polymerized polyphenol. Likewise, a high correspondence can be observed between the SERS spectra of GA and TA (Figure 5b,d), the main difference being the existence of a medium band at 1704 cm^−^^1^ attributed to the ester ν(C–O) vibration, which seems to be absent in the SERS spectrum of GA. This similarity corroborates that GA undergoes a polymerization on the surface, although it does not involve the formation of ester groups.

## 3. Materials and Methods

### 3.1. Materials

Pyrogallol (1,2,3-trihydroxybenzene; CAS Number: 87-66-1; PY), gallic acid (3,4,5-trihydroxybenzoic acid; CAS Number: 149-91-7; GA), tannic acid (CAS Number: 1401-55-4; TA), and syringic acid (3,5-dimethoxy-4-hydroxybenzoic acid; CAS Number: 530-57-4; SA) were purchased from Sigma-Aldrich and used as such for the spectral measurements. Stock solutions of polyphenols in water were prepared at a concentration of 20 mg/mL and stored in the dark to protect them from light [70]. For the same reason and in order to minimize the possible photodegradation of phenol molecules, the examined solutions were protected from light during out-of-measurement times.

Silver nitrate, trisodium citrate dihydrate, hydroxylamine hydrochloride, and other reagents were of analytical grade and purchased from Sigma-Aldrich and Fluka. All solutions were freshly prepared with Milli-Q water before the experiments and used immediately.

### 3.2. Spectroscopic Measurements

The UV-Vis absorption spectra were recorded at room temperature employing a Shimadzu UV-2401 spectrophotometer over the range of 200–900 nm and using 1 cm-path-length quartz cuvettes.

Fourier transform infrared (FTIR) spectra of pure polyphenols in the powder forms were recorded in attenuated total reflectance (ATR) mode at room temperature in the region of 2000–520 cm^−^^1^ managed on the FTIR spectrometer (ABB, Model FTLA2000-100) using a Nicolet 8700 IR Microscope. The spectra resolution was set to 4 cm^−^^1^, and the final spectra were the results of 128 scans.

Fourier transform Raman (FT-Raman) spectra were obtained by using a Bruker RFS 100/S spectrometer. Radiation of 1064 nm from an air-cooled Nd: YAG laser was used for excitation. The resolution was set to 4 cm^−^^1^, and a 180° geometry was employed. The output laser power was 200 mW, and the laser power at the sample was 20 mW. The Raman spectra displayed in this work were the result of averaging 1000 accumulations.

The dispersive Raman and SERS spectra were obtained with a confocal Raman microspectrometer Renishaw InVia equipped with an electrically refrigerated CCD camera and coupled to a Leica microscope. The excitation source used with this instrument was a 785 nm laser line. The Raman signal was collected over the range of 100–2000 cm^−^^1^, under macro conditions using glass vials and working with a spectral resolution of 1 cm^−^^1^. The laser power at the sample was up to 2 mW. The signal was firstly calibrated by using the 520 cm^−^^1^ line of a Si wafer and a 20× objective.

SERS measurements were carried out on silver colloids. Ag nanoparticles (NPs) were prepared by the reduction of silver nitrate with hydroxylamine hydrochloride following Leopold and Lendl’s method [71] (AgH colloid) or with trisodium citrate dihydrate by Lee and Meisel’s method [72] (AgC colloid). As default, the pH value, UV-Vis extinction, and Raman spectra of such colloids were checked before their employment. The prepared AgC and AgH colloids demonstrated the common characteristics required for such SERS substrates [66,73]. Since the addition of the polyphenol molecule did not induce any significant aggregation of the colloid, the activation of the colloid before adding the polyphenols was required. This activation consisted of a partial aggregation of the colloidal particles, and to accomplish this, an aliquot (usually 50 μL) of 0.5 M potassium nitrate was added to 1 mL of the colloid. The final concentration of the polyphenols in the colloidal suspensions was 10^−^^5^ M.

## 4. Conclusions

The FT-Raman, FTIR, and SERS spectra of the gallnut polyphenols tannic acid, gallic acid, pyrogallol, and syringic acid displayed many similarities and differences that allowed performing a differential assignation of the bands and finding specific marker features that could identify these compounds in complex polyphenol mixtures. The different functional groups existing in these molecules and their spatial distribution led to slight changes of the vibrations. The Raman spectra were dominated by bands corresponding to the ring vibrations, but the substituents in the ring strongly affected these vibrations. In pyrogallol, a strong ring-breathing band was observed, but this band was absent in the other carboxylic acid-containing polyphenols. The existence of methoxy –OCH_3_ groups in syringic acid displayed characteristic bands that were absent in the other compounds.

The FTIR spectra of these molecules were dominated by the peripheral oxygen-containing substituents of the aromatic ring, displaying strong broad bands in the range of 1200–1000 cm^−^^1^ attributed to ν(C–O), ν(C–C), and in-plane δ(C–H), which converted this region into an actual fingerprint of these molecules. Furthermore, the out-of-plane bending vibrations of the O–H group revealed the existence of characteristic marker bands associated with each polyphenol.

The SERS spectra of the analyzed polyphenols showed significant changes in comparison with the Raman spectra. This result indicated the occurrence of strong polyphenol structural modifications as a consequence of their interaction with the metal surface. Another effect observed in the SERS spectra was the broadening of the bands derived from a possible polymerization of these molecules to more complex structures. The most important modifications observed in the SERS spectra of these compounds were the remarkable weakening of the *8a* ring vibration and the subsequent intensification of the *19a* mode of the benzene ring. This mode was also more intense in the FTIR spectra, and its intensification in the SERS spectra could be related to an increase of the variation of the polarizability associated with the *19a* mode as a consequence of the structural change of the molecule induced by the strong interaction with the metal in the Ag NPs.

Tannic acid was the polyphenol that displayed a lower difference in comparison to the corresponding Raman spectrum, and this was attributed to a weaker interaction with the surface, which implied a lower structural modification when adsorbed on the surface. In contrast, gallic acid underwent deep structural changes on the Ag surface. The similarity of the SERS spectrum of gallic acid with that of tannic acid suggested that the first may undergo a polymerization when adsorbed on the surface of silver.

## Figures and Tables

**Table 1:** Observed (IR and Raman; solid state, λ_exc_ = 1064 nm) vibrational frequencies of the studied polyphenol molecules (pyrogallol, PY; gallic acid, GA; tannic acid, TA; syringic acid, SA).

FTIR ^a^/cm^−1^				FT-Raman ^a^/cm^−1^				Vibrational Assignment ^b,c^
PY	GA	TA	SA	PY	GA	TA	SA	
				157 *w*	142 *w*		149 *w-m, sh*	δ(CCC)
				184 *vw*			178 *w-m*	
				198 *vw*			207 *w*	
					219 *vw*		221 *vw, sh*	
					242 *vw*			
				271 *w-m*		269 *w*		
							296 *w*	
				321 *w*	317 *vw*			γ(CO), γ(ring)
				337 *w*		331 *vw, sh*		
					360 *w-m*	360 *w*	362 *w-m, sh*	δ(CO)
					389 *w-m*		385 *m*	
					437 *w*			γ(OH), γ(ring)
					454 *w, sh*			
							485 *w*	
				504 *w, sh*				γ(ring)
				522 *w*		514 *w*		
540 *w*	532 *w-m*			545 *vw, sh*	547 *w-m, sh*		545 *w*	
	**555 *s***				554 *w-m*	551 *w*		
582 *m*		586 *vw, br*	577 *m-s*	583 *vw*	572 *w, sh*		587 *w*	γ(CO), γ(ring), *16a*
				597 *vw*				γ(CO), ν(ring)
	635 *m*							
	648 *w-m, sh*	650 *w, br*				653 *vw*		
669 *m, sh*	669 *m*	669 *w*	**669 *m-s***				672 *vw, sh*	γ(OH)
**700 *vs***	692 *m, br*		**687 *m-s***		695 *vw, sh*	693 *vw*	689 *w-m*	
715 *m*				714 *s*	**724 *w***			ν(ring breathing)
**764 *s***	**731 *s-vs***	**737 *m, sh***	737 *w*		740 *vw, sh*	753 *w-m*	738 *vw*	γ(CH) + γ(OH)
	766 *m*	**754 *m-s***	**768 *m-s***		774 *w*	786 *w-m*		
	791 *w*	781 *w, sh*						γ(CH)
	800 *w*		802 *w*		803 *w*		803 *m*	
827 *m*		831 *w, sh*	841 *vw*	830 *w*		830 *w*	844 *vw*	
860 *vw*	864 *s*		864 *w-m*	857 *vw*	850 *vw*			
872 *w*		870 *m*			877 *vw*	878 *vw*	867 *vw*	
	899 *m, br*		908 *w-m*				909 *w*	
	959 *w*	957 *m, sh, br*	937 *w*	944 *vw*	963 *w-m*	959 *w*	938 *w*	ν(C–COOH)
**999 *vs***		**1014 *s-vs***		1008 *vw*		1019 *vw*		δ(CH), ν(ring), *18a*
	**1022 *vs***		1040 *m*		1037 *vw*		1033 *w-m*	
1065 *m*				1065 *m*				δ(CH)
	1097 *w*	1082 *m-s*		1091 *w, sh*	1091 *w, sh*	1091 *w*		δ(CH), *9b*
			**1101 *vs***		1102 *w*		1102 *w-m*	δ(CH), ν(CO)
							1116 *w, sh*	
1157 *s*				1156 *m*			1154 *w*	
	1178 *s-vs*	**1178 *vs, br***	**1175 *s***		1174 *vw*		1179 *s, sh*	δ(CH), δ(OH)
**1190 *vs***	1202 *s-vs*		**1194 *s***	1202 *w*		**1200 *m***	1199 *s*	
	1217 *s-vs*				1220 *w*			δ(CH), ν(ring), ν(CO)
1242 *s*	1243 *m*		1244 *w-m*	1241 *vw*			1239 *w*	
1283 *s*	1261 *s-vs*		1265 *w*	1276 *w*	**1266 *m-s***	1260 *w-m, sh*	1266 *w-m*	
				1293 *vw, sh*				
1317 *s*	1309 *s*	1308 *s-vs*	1317 *m*		1324 *m-s*	1322 *m*	1322 *m*	δ(CH), δ(OH), δ(C–OH),ν(ring)
1329 *s, sh*				1343 *w-m*	1334 *m-s, sh*			
1350 *s, sh*			1367 *m-s*		1368 *m*	**1370 *m***	1370 *m*	
1385 *s, sh*	1383 *w*		1383 *m, sh*	1384 *w*			1386 *w, sh*	
1398 *s, br*								
			1418 *m*				1424 *w*	ν(ring), δ(OH)
		1444 *m*			1443 *w*	1447 *w*	1445 *w*	
			1456 *m*			1466 *vw, sh*	1469 *w*	ν(ring), δ(OH), *19a*
1481 *s*				1490 *w*	1484 *vw*			
1518 *s*		1533 *m, br*	1520 *m*	1528 *w*	**1530 *w-m***	1536 *vw*	1521 *vw*	ν(ring), δ(C–OH),δ(CH), *19b*
	1541 *m*							
			1595 *m, sh*		1598 *vs*		1594 *vs*	ν(ring), δ(OH), δ(CH), *8a*
**1618 *s***	1608 *s*	1606 *s*	1616 *m*	1627 *m*	1615 *vs*	1615 *vs*		
							1658 *vw*	
	1672 *m, br*	**1699 *s, br***	1693 *m-s*		**1692 *w-m***		1698 *vs*	ν(C=O) in acid
						**1711 *s***		ν(C=O) in ester

^a^***vw***, very weak; ***w***, weak; ***w-m***, weak medium; ***m***, medium; ***m-s***, medium strong; ***s***, strong; ***s-vs***, strong very strong; ***vs***, very strong; ***sh***, shoulder; ***d***, doublet; ***br*** – broad; ^b^ **ν**, stretching; **δ**, in-plane bending/deformation; **γ**, out-of-plane bending; ^c^ The assignments were made on the basis of references [49,56,58,59,60,61,62].

## Data Availability

Not applicable.
